# Role of Myeloperoxidase Oxidants in the Modulation of Cellular Lysosomal Enzyme Function: A Contributing Factor to Macrophage Dysfunction in Atherosclerosis?

**DOI:** 10.1371/journal.pone.0168844

**Published:** 2016-12-20

**Authors:** Fahd O. Ismael, Tessa J. Barrett, Diba Sheipouri, Bronwyn E. Brown, Michael J. Davies, Clare L. Hawkins

**Affiliations:** 1 The Heart Research Institute, Sydney, New South Wales, Australia; 2 Sydney Medical School, University of Sydney, Sydney, New South Wales, Australia; 3 Department of Biomedical Sciences, Panum Institute, University of Copenhagen, Copenhagen, Denmark; Hospital for Sick Children, CANADA

## Abstract

Low-density lipoprotein (LDL) is the major source of lipid within atherosclerotic lesions. Myeloperoxidase (MPO) is present in lesions and forms the reactive oxidants hypochlorous acid (HOCl) and hypothiocyanous acid (HOSCN). These oxidants modify LDL and have been strongly linked with the development of atherosclerosis. In this study, we examined the effect of HOCl, HOSCN and LDL pre-treated with these oxidants on the function of lysosomal enzymes responsible for protein catabolism and lipid hydrolysis in murine macrophage-like J774A.1 cells. In each case, the cells were exposed to HOCl or HOSCN or LDL pre-treated with these oxidants. Lysosomal cathepsin (B, L and D) and acid lipase activities were quantified, with cathepsin and LAMP-1 protein levels determined by Western blotting. Exposure of J774A.1 cells to HOCl or HOSCN resulted in a significant decrease in the activity of the Cys-dependent cathepsins B and L, but not the Asp-dependent cathepsin D. Cathepsins B and L were also inhibited in macrophages exposed to HOSCN-modified, and to a lesser extent, HOCl-modified LDL. No change was seen in cathepsin D activity or the expression of the cathepsin proteins or lysosomal marker protein LAMP-1. The activity of lysosomal acid lipase was also decreased on treatment of macrophages with each modified LDL. Taken together, these results suggest that HOCl, HOSCN and LDL modified by these oxidants could contribute to lysosomal dysfunction and thus perturb the cellular processing of LDL, which could be important during the development of atherosclerosis.

## Introduction

The uncontrolled uptake of modified low-density lipoprotein (LDL) by macrophage scavenger receptors results in lipid accumulation and “foam cell” formation, and is a key event in the development of atherosclerosis [1, 2]. The uptake of native LDL occurs via feedback-controlled receptor-mediated endocytosis, whereas modified LDL uptake occurs in a non-controlled manner via multiple scavenger receptors. In both cases, the LDL is transported via the endosomal system to lysosomes [3, 4]. Modified LDL can also be delivered to lysosomes by macrophage autophagy [5]. Lysosomes are rich in cathepsin proteases and an ester hydrolase, lysosomal acid lipase (LAL), which work together to metabolise native LDL, and detoxify modified LDL [6]. Changes in lysosome function have been observed early in disease pathology [5, 7], with ineffective lysosomal degradation of modified LDL postulated to be a key pathway in the accumulation of modified / dysfunctional proteins, cholesterol and lipid within the arterial wall [3, 8].

LDL can be modified by multiple pathways to give pro-atherogenic particles, with the term “oxLDL” used to describe different LDL preparations that have been modified *ex vivo* or isolated from biological material, which have specific fingerprints of oxidation and reactivity [9]. LDL modified by exposure to Cu^2+^ is both resistant to degradation by lysosomal cathepsins, and induces the inactivation of the Cys-dependent cathepsin enzymes B and L, which together contributes to the macrophage accumulation of modified LDL [3, 8, 10–12]. However, the relevance of LDL modified by Cu^2+^ to human disease has been questioned (reviewed [2]), primarily because the concentration of Cu^2+^ present in even the most advanced lesions (≈7.5 nM) is orders of magnitude lower than the concentration commonly used to prepare oxLDL *ex vivo* [13].

Human atherosclerotic lesions contain increased amounts of myeloperoxidase (MPO) [14], a heme enzyme released by activated phagocytes that produces the chemical oxidants hypochlorous acid (HOCl) and hypothiocyanous acid (HOSCN) [15]. Although these oxidants have an important immune function by killing invading pathogens and preventing bacterial cell growth, the overproduction of MPO-derived oxidants in the vessel wall during chronic inflammation is strongly implicated in atherosclerosis [4, 15]. These data are supported by several epidemiological studies showing clear association between MPO and the development of atherosclerosis and as a prognostic agent to predict patient outcome following chest pain and major cardiovascular events (reviewed [16]). The detection of increased levels of the HOCl-specific marker, 3-chlorotyrosine, in LDL isolated from human lesions [17, 18], together with evidence for the presence of MPO-LDL complexes in the circulation of patients with atherosclerosis [19], support MPO as a pathway to LDL modification *in vivo*.

The modification of LDL by HOCl has potent pro-atherogenic effects, including promoting macrophage cholesterol accumulation and endothelial dysfunction (reviewed [4]). The role of HOCl-modified LDL on lysosomal function has not been examined in detail, though this type of oxLDL can inhibit isolated cathepsin B in a non-cellular environment [20]. In contrast, the role of HOSCN in the modification of LDL and development of atherosclerosis is unclear. A correlation between serum levels of thiocyanate (SCN^-^), the precursor to HOSCN, with higher macrophage foam cell populations [21] and fatty streak formation [22] in smokers supports a role for this oxidant in disease pathology. However, macrophages exposed to HOSCN-modified LDL accumulate cholesterol to a lesser extent compared to HOCl-modified LDL [19, 23], and human MPO transgenic atherosclerosis-prone mice supplemented with SCN^-^ show a reduced extent of lesion formation [24].

In light of these conflicting data, we examined the reactivity of each oxidant directly, and HOCl and HOSCN-modified LDL on the activity of lysosomal enzymes within macrophages [5, 7]. The effect of HOSCN-modified LDL on the activity of the lysosomal cathepsin enzymes (B, L and D) and LAL was compared to LDL exposed to HOCl and cyanate (OCN^-^), which is a decomposition product of HOSCN that has also been implicated in atherogenesis [25]. This is important because HOCl, HOSCN and OCN^-^ have different fingerprints of LDL modification and hence biological reactivity [23], which differ from that seen on exposure of LDL to Cu^2+^ [26, 27].

## Materials and Methods

### Reagents

All aqueous reagents were prepared using nanopure water, filtered through a four stage Milli-Q system. HOCl was prepared by dilution of a concentrated stock solution of NaOCl (Merck) into PBS. HOSCN was enzymatically prepared using lactoperoxidase (LPO; from bovine milk; Calbiochem) [28], and used immediately after quantification with 5-thio-2-nitrobenzoic acid (TNB; Sigma-Aldrich), with a molar absorption coefficient of 14,150 M^−1^cm^−1^ at 412 nm [29, 30].

### Low-density lipoprotein isolation and modification

Plasma was isolated from healthy donors with informed, written, consent and approval from the Sydney Local Health District Ethics Committee (Sydney Local Health District; Protocol X09-0013 and X12-0375). LDL (1.019 < d < 1.06 g/ml) were isolated as previously described [23, 31]. The protein concentration of isolated LDL was assessed using the bicinchoninic acid (BCA) assay. Stock solutions of LDL were purified immediately before treatment using a PD-10 column (GE Healthcare) and diluted to the required concentration (1 mg mL^-1^ based on apoB100) into Chelex-treated PBS. LDL was exposed HOSCN or HOCl (0–500 μM) for 30 min at 22°C or 24 h at 37°C or KOCN (0–5000 μM) for 24 h at 37°C. Any residual, unreacted excess oxidant was removed using a PD-10 column.

### Tissue culture

The J774A.1 murine macrophage-like cells (ATCC: TIB-67) were grown in Dulbecco’s modified Eagle’s medium (DMEM; JRH Biosciences) supplemented with 10% (v/v) Fetal Bovine Serum, 100 U mL^-1^ penicillin and 0.1 mg mL^-1^ streptomycin (Invitrogen) and 2 mM L-glutamine (Thermotrace), in 175 cm^2^ tissue culture flasks at 37°C in a humidified atmosphere of 5% CO_2_. Prior to all experiments, confluent J774A.1 cells were seeded overnight at a density of 0.5 x 10^6^ cells per well in 6 or 12-well culture plates (Costar). For LDL incubations, 10% (v/v) lipoprotein deficient serum replaced Fetal Bovine Serum.

### Treatment of intact cells and lysates with oxidants or modified LDL

For the lysate experiments, cells were washed, pelleted and lysed in 500 μL of water for 30 min at 4°C, followed by 3 repeated cycles of freeze-thawing. Cell debris was removed by centrifugation at 2000 *g* for 5 min at 4°C. Lysates were then treated with HOSCN (0–20 μM) for 15 min at 22°C, followed by incubation with DTT (100 μM) and assaying enzyme activity. For LDL experiments, the LDL was modified as described above, before addition to the lysates for 15 min at 22°C. For intact cell experiments, cells were washed prior to exposure to HOSCN or HOCl (0–160 μM) for 15 min at 22°C or each type of modified LDL for 4 and 24 h.

### Lysosomal enzyme activity assays and protein expression

Cathepsin B, L and D activities were assessed fluorometrically using the following substrates: Z-Arg-Arg-AMC (Bachem, Bulbendorf, Switzerland), Z-Phe-Arg-AMC (Bachem) and 7-methoxycoumarin-4-acetyl-Gly-Lys-Pro-Ile-Leu-Phe-Phe-Arg-Leu-Lys-DNP-D-Arg-amide, as described previously [32]. LAL was determined by using the pro-fluorescent substrate 4-methylumbelliferyl oleate [33]. Western blotting was used to assess changes in the protein expression of cathepsin B, L and LAMP-1, following lysis of the cells in water at 4°C, and electrophoresis (90 min, 130 V) using 4–12% bis-tris gels (Novex Nupage system, Life Technologies, Carlsbad, CA, USA) with protein transfer (20 V, 7 min) to a PDVF membrane (iBlot 2, Life Technologies). And incubation with either anti-cathepsin B goat polyclonal (Santa Cruz Biotechnology, Dallas, Texas, USA), anti-cathepsin L mouse monoclonal (Abcam, Cambridge, UK), anti-LAMP-1 rabbit polyclonal (Abcam) or anti-β-actin mouse monoclonal (Santa Cruz) primary antibodies (1/1000 dilution). Proteins were visualized using a ChemiDoc XRS (Bio-Rad, Hercules, CA, USA), following exposure of membranes to chemiluminescence reagents (Western Lightening Plus-ECL, Perkin Elmer, Waltham, MA, USA), with densitometry performed using ImageJ software (National Institutes of Health, USA).

### Statistical analyses

Data are expressed as mean ± SEM from at least 3 independent experiments, with LDL from at least 3 different donors. Statistical analyses were performed using 1-way ANOVA with Tukey’s post-hoc testing or 2-way ANOVA with Bonferroni’s post-hoc testing (GraphPad Prism 6, GraphPad Software, San Diego, USA), with p < 0.05 taken as a significant.

## Results

### HOCl and HOSCN inactivate lysosomal cathepsin enzymes in J774A.1 macrophages

**Fig 1:**
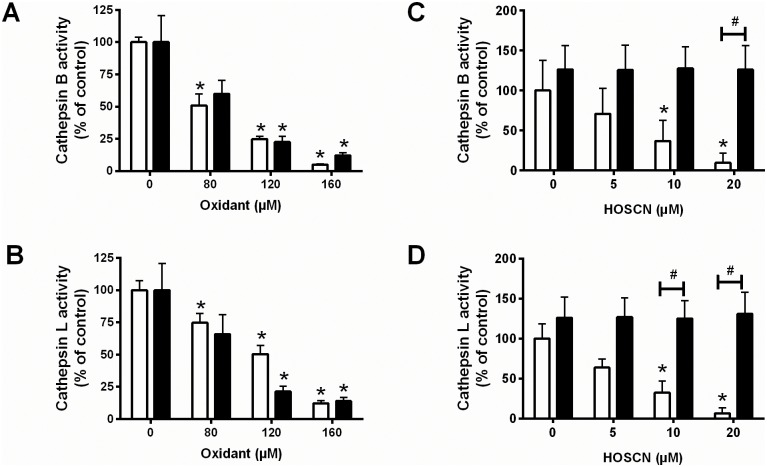
Inhibition of cathepsin B and L activity in J774A.1 cells after treatment with HOCl and HOSCN. (A) Cathepsin B and (B) cathepsin L activity in J774A.1 cells (1 × 10^6^ cells mL^-1^) was determined after incubation with HOSCN (80–160 μM, white bars) or HOCl (80–160 μM, black bars) for 15 min at 22°C. (C) Cathepsin B and (D) cathepsin L activity in J774A.1 cell lysates (1 × 10^6^ cells mL^-1^) after incubation with HOSCN (5–20 μM) for 15 min, followed by further incubation in the absence (white bars) or presence (black bars) of DTT (100 μM) for 15 min. Results are expressed as a percentage of the PBS-treated control cells. * and # represent a significant (p < 0.05) change in cathepsin B/L activity compared with control lysates or the presence / absence of DTT, respectively.

Exposure of intact J774A.1 cells to HOCl or HOSCN (80–160 μM) for 15 min resulted in a dose-dependent loss in the enzymatic activity of both cathepsin B (Fig 1A) and cathepsin L (Fig 1B). In each case, the extent of enzyme inactivation observed with HOSCN was comparable to that with HOCl (Fig 1). No loss in cell viability was observed under these treatment conditions, in accord with previous studies [34]. The mechanism involved in cathepsin inactivation was assessed in experiments with lysates treated with HOSCN (5–20 μM), where almost complete inhibition of both enzymes was observed with 20 μM HOSCN (Fig 1C and 1D, white bars). The loss in cathepsin activity was reversed to levels comparable to the non-treated control cells on adding the reducing agent DTT (100 μM; Fig 1C and 1D, black bars). This is consistent with the formation of Cys-derived, reversible oxidation products. In contrast, no change in activity was observed in the corresponding experiments with the Asp-dependent cathepsin D on treatment of J774A.1 lysates with HOSCN (25–250 μM) (data not shown).

### Perturbation of lysosomal cathepsin activity but not expression in J774A.1 cells exposed to modified LDL

**Fig 2:**
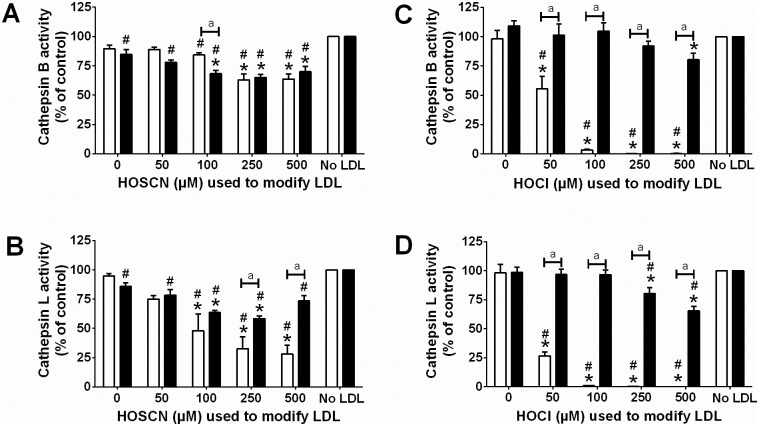
Inhibition of cathepsin B and L activity after exposure of J774A.1 cell lysates to HOSCN- and HOCl-modified LDL. LDL (1 mg protein mL^-1^) was exposed to 0–500 μM HOSCN (A and B) or HOCl (C and D) for 30 min (white) and 24 h (black) at 22°C and 37°C respectively, prior to addition of each modified LDL (0.1 mg protein mL^-1^) to J774A.1 lysates for 15 min at 22°C, followed by determination of cathepsin B (A, C) or cathepsin L (B, D) activity, which is expressed relative to the no LDL control. Data are means ± SEM for at least 3 independent experiments, with multiple LDL donors. * and # represent a significant (p < 0.05) decrease in cathepsin B or L activity compared to cells exposed to the incubation control LDL or no LDL. “a” represents a significant (p < 0.05) difference between 30 min and 24 h modified LDL.

**Fig 3:**
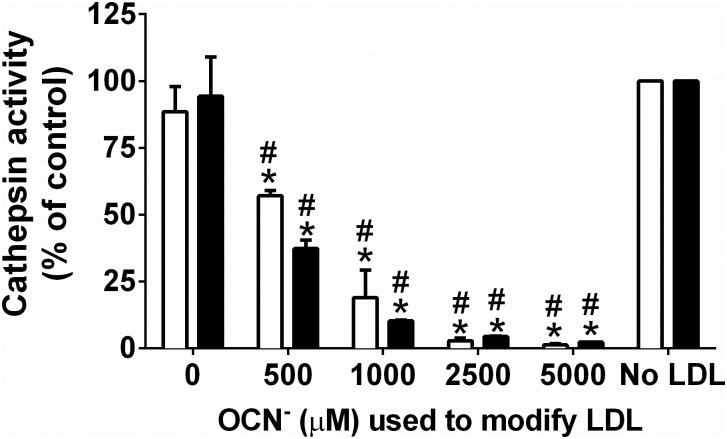
Inhibition of cathepsin B and L activity after exposure of J774A.1 cell lysates to OCN^-^modified LDL. LDL (1 mg protein mL^-1^) was exposed to 0–5000 μM KOCN for 24 h at 37°C, prior to addition of each modified LDL (0.1 mg protein mL^-1^) to J774A.1 lysates for 15 min at 22°C, followed by determination of cathepsin B (white bars) or cathepsin L (black bars) activity, which is expressed relative to the no LDL control. * and # represent a significant decrease (p < 0.05) in cathepsin B or L activity compared with cells exposed to the incubation control LDL or no LDL respectively by 1-way ANOVA with Tukey’s post-hoc testing.

Exposure of J774A.1 lysates to LDL (1 mg mL^-1^) pre-treated (for 30 min or 24 h) with HOSCN (0–500 μM) for 15 min at 22°C resulted in a significant HOSCN concentration-dependent decrease in the activity of cathepsin B (Fig 2A) and cathepsin L (Fig 2B). For cathepsin B, a similar extent of inactivation was observed with LDL pre-treated with HOSCN for 30 min or 24 h (Fig 2A). With cathepsin L, a greater extent of inactivation was observed on pre-treatment of the LDL with HOSCN for 30 min rather than 24 h (Fig 2B, white versus black bars). Treatment of J774A.1 lysates with HOCl-modified LDL also resulted in a significant HOCl concentration-dependent decrease in cathepsin B and L activities. In each case, this loss in activity was more marked with the 30 min pre-treatment of LDL rather than the 24 h pre-treatment (Fig 2C and 2D, white versus black bars). Addition of Met (20 mM) to the HOCl-treated LDL to quench *N*-chloramines (and other reactive species), before reaction with the cell lysates prevented the inactivation of cathepsin B and L (data not shown). Inactivation of cathepsin B and L enzymes was also observed after exposure of J774A.1 cell lysates to LDL pre-treated with the HOSCN decomposition product OCN^-^ (0–5 mM) for 24 h prior to addition to the cells (Fig 3).

**Fig 4:**
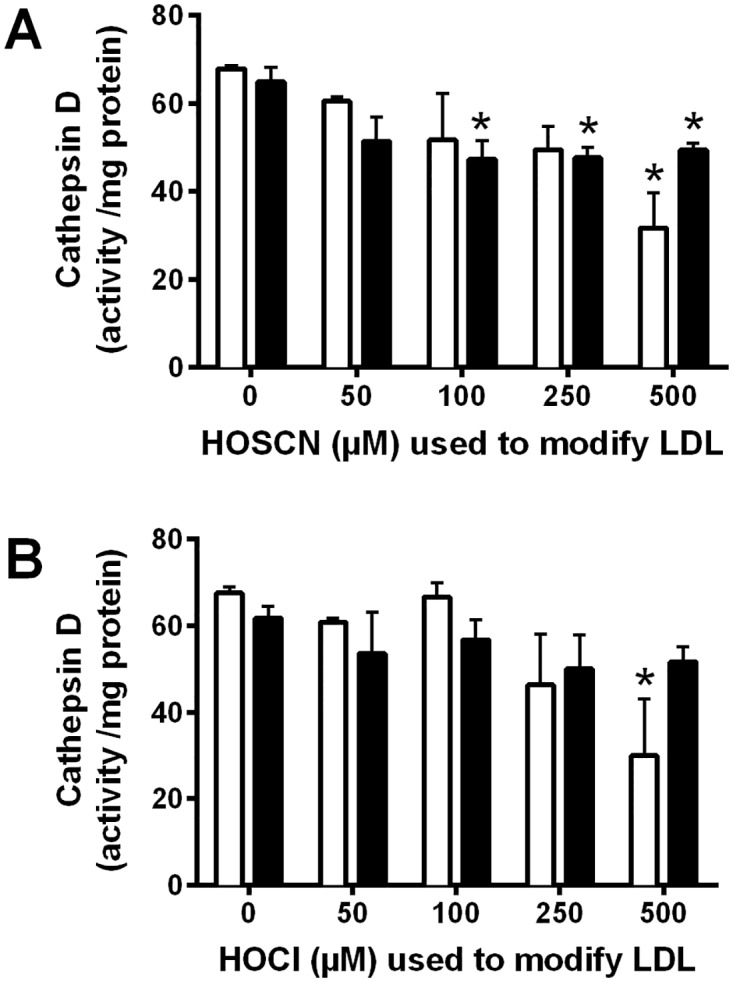
Inhibition of cathepsin D activity after exposure of J774A.1 cell lysates to HOSCN- and HOCl-modified LDL. LDL (1 mg protein mL^-1^) was exposed to 0–500 μM HOSCN (A) or HOCl (B) for 30 min (white) and 24 h (black) at 22°C and 37°C, respectively, prior to addition of each modified LDL (0.1 mg protein mL^-1^) to J774A.1 lysates for 15 min at 22°C, followed by determination of cathepsin D activity expressed as activity/mg protein. * represents a significant decrease (p < 0.05) in cathepsin D activity compared with cells exposed to the incubation control LDL. There was no significant difference in enzyme inhibition between 30 min and 24 h LDL oxidant, as determined by 2-way ANOVA.

Inactivation of the Asp-dependent cathepsin D was observed after exposure of J774A.1 lysates to HOSCN-modified LDL in experiments where the LDL was pre-treated with ≥ 100 μM of HOSCN (Fig 4A). In contrast, a loss in cathepsin D activity in cell lysates was only seen with HOCl-modified LDL under conditions where LDL was pre-treated with 500 μM HOCl for 30 min, which may be related to the formation of high levels of *N*-chloramines (Fig 4B). Cathepsin D enzyme activity was unaffected when J774A.1 lysates were exposed to LDL pre-treated with up to 2500 μM OCN^-^ (data not shown).

**Fig 5:**
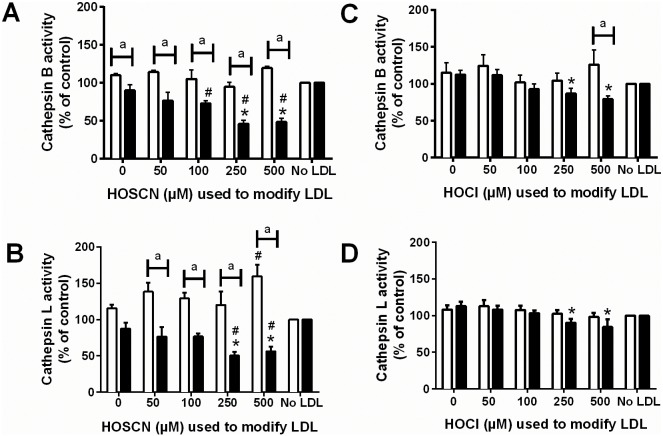
Inhibition of cathepsin B and L activity after exposure of intact J774A.1 cells to HOSCN- and HOCl-modified LDL. LDL (1 mg protein mL^-1^) was exposed to 0–500 μM HOSCN (A and B) or HOCl (C and D) for 24 h at 37°C respectively, prior to addition of each modified LDL (0.1 mg protein mL^-1^) to J774A.1 cells for 4 (white bars) or 24 h (black bars), and determination of cathepsin B (A, C) or cathepsin L (B, D) activity, which is expressed relative to the no LDL control. * and # represent a significant decrease (p < 0.05) in cathepsin B or L activity compared with cells exposed to the incubation control LDL or no LDL. “a” represents a significant (p < 0.05) difference between cathepsin activity between cells incubated with LDL for 4 or 24 h.

In contrast, there were no significant changes in cathepsin B or L activities when intact J774A.1 cells were exposed to LDL pre-treated for 30 min with HOSCN or HOCl for 4 or 24 h (data not shown). This may reflect quenching of *N*-chloramines and other reactive species by cell media components, which were not present in the cell lysate experiments, or the presence of an intact cell membrane. However, when J774A.1 cells were exposed to LDL modified with HOSCN for 24 h, significant HOSCN concentration-dependent decreases in cathepsin B (Fig 5A) and L (Fig 5B) activities were detected after 24 h (black bars) but not 4 h (white bars) incubation with the cells. With cells exposed to HOCl-modified for 24 h, significant decreases in cathepsin B (Fig 5C) and L (Fig 5D) were also observed, though to a lesser extent than compared to HOSCN-modified LDL. LDL pre-treated with up to 2500 μM OCN^-^ before incubation with cells for 24 h, did not significantly affect cathepsin B or L activities (data not shown).

**Fig 6:**
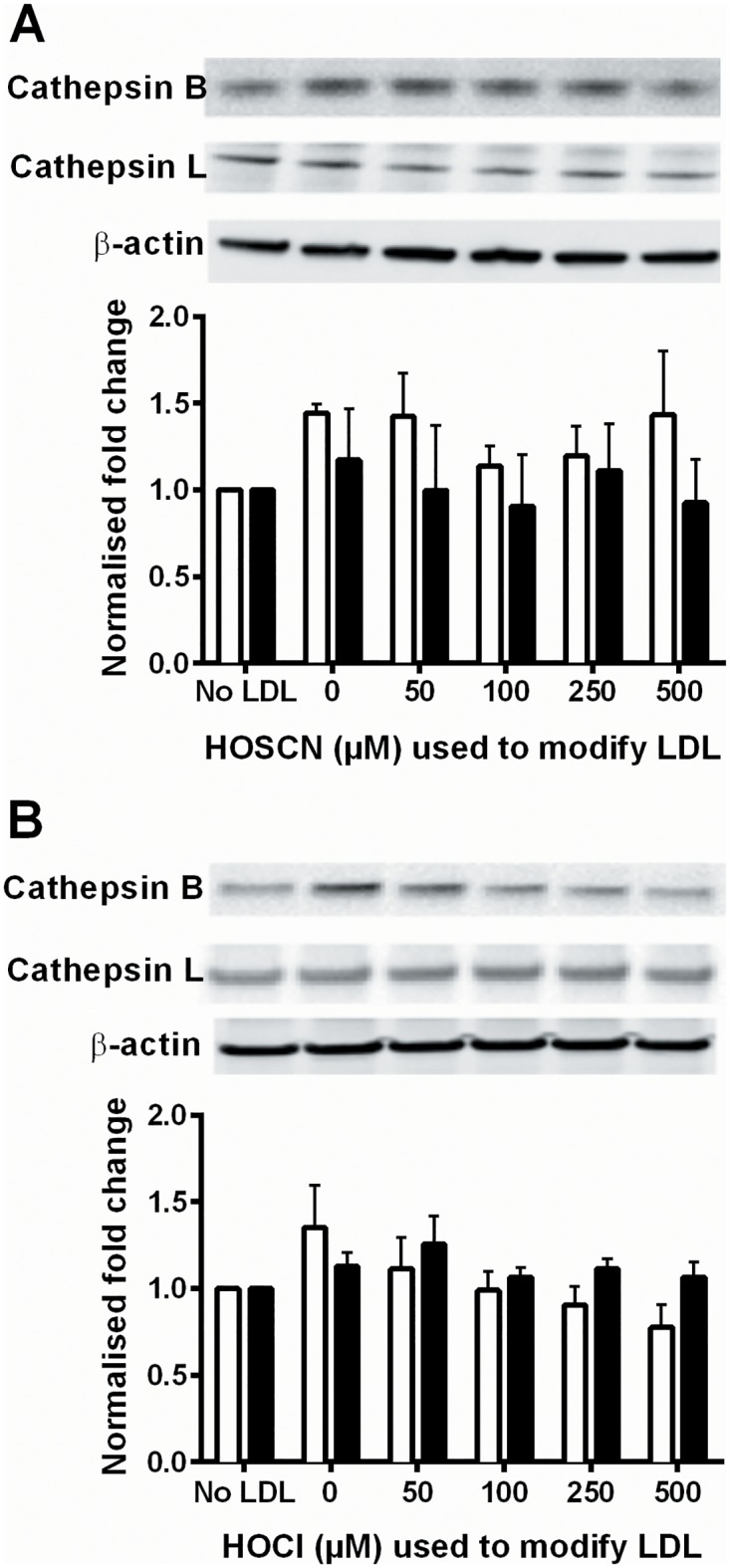
Cathepsin B and L protein levels are unchanged in J774A.1 cells after exposure to HOSCN- and HOCl-modified LDL. LDL (1 mg protein mL^-1^) was exposed to 0–500 μM HOSCN (A) or HOCl (B) for 24 h at 37°C, respectively, prior to addition of each modified LDL (0.1 mg protein mL^-1^) to J774A.1 cells, followed by determination of cathepsin B (white) and L (black) protein expression. Cathepsin levels were normalised to β-actin levels, and then calculated as the fold change from the no LDL condition. Representative blots of the cathepsin B band at 25 kDa, the cathepsin L band at 25 kDa, or the β-actin band at 43 kDa, are displayed, of n = 3–4 separate experiments. There was no significant effect of oxidant treatment as determined by 1-way ANOVA on either cathepsin protein levels.

**Fig 7:**
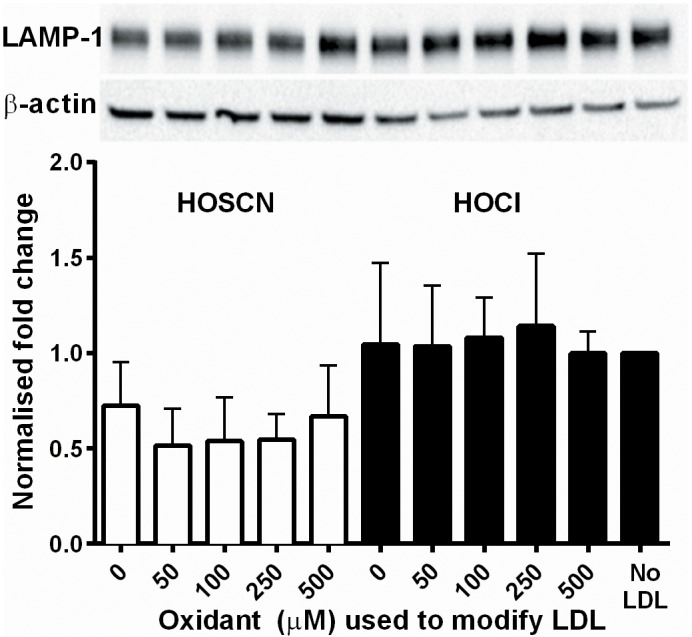
LAMP-1 protein levels are unchanged in J774A.1 cells after exposure to HOSCN- and HOCl-modified LDL. LDL (1 mg protein mL^-1^) was exposed to 0–500 μM HOSCN (white) or HOCl (black) for 24 h at 37°C prior to addition of each modified LDL (0.1 mg protein mL^-1^) to J774A.1 cells, followed by determination of LAMP-1 protein expression. LAMP-1 levels were normalised to β-actin levels, and then calculated as the fold change from the no LDL condition. Representative blots of the LAMP-1 band at 120 kDa, or the β-actin band at 43 kDa, are displayed, of n = 3 separate experiments. There was no significant effect of oxidant treatment as determined by 1-way ANOVA on LAMP-1 levels.

The changes in cathepsin enzyme activity were not related to altered protein expression or lysosomal number, no significant changes in the protein levels of cathepsins B and L (Fig 6), or the lysosomal marker protein LAMP-1 (Fig 7) were detected on treatment of J774A.1 cells for 24 h with LDL modified by HOSCN or HOCl. In addition, there was no significant change in the activity of cathepsin D on treatment of macrophages with LDL modified by HOCl, HOSCN or OCN^-^.

### Modified LDL induces inactivation of lysosomal acid lipase in J774A.1 macrophages

**Fig 8:**
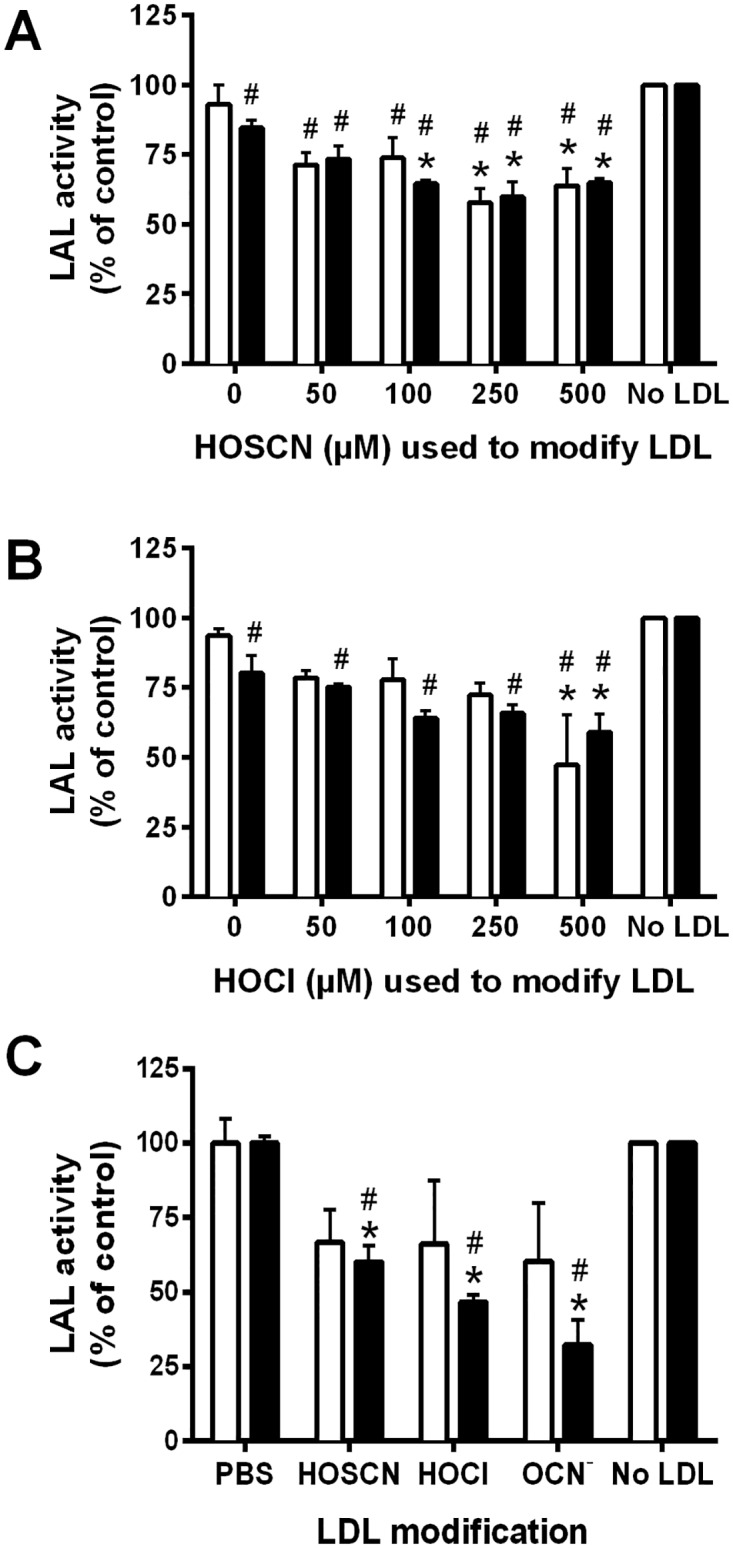
Inhibition of lysosomal acid lipase (LAL) activity after exposure of J774A.1 lysates and cells to modified LDL. LDL (1 mg protein mL^-1^) was exposed to (A) HOSCN (0–500 μM) or (B) HOCl (0–500 μM) for 30 min (white) and 24 h (black) at 22°C and 37°C, respectively, prior to addition of each modified LDL (0.1 mg protein mL^-1^) to J774A.1 lysates for 15 min at 22°C, and determination of LAL activity, which is expressed relative to the no LDL control. Graph (C) shows LAL activity after exposure of J774A.1 cells for 4 (white) or 24 h (black) at 37°C to LDL modified by HOSCN (250 μM), HOCl (250 μM), or OCN^-^ (2500 μM) for 24 h at 37°C. * and # represent a significant decrease (p < 0.05) in LAL activity compared with cells exposed to the incubation control LDL or no LDL.

LAL is the sole lysosomal enzyme responsible for hydrolyzing endocytosed cholesteryl esters and triglycerides [35]. No significant decrease in LAL activity was seen on pre-treatment of LDL with HOSCN for 30 min prior to addition to cell lysates. However, incubation of cell lysates with LDL pre-treated with > 100 μM HOSCN for 24 h resulted in a significant decrease in LAL activity (Fig 8A). A decrease in LAL activity was also observed in analogous experiments performed with LDL pre-treated with HOCl, though in this case, a significant loss of activity was only observed with the 500 μM treatment condition (Fig 8B). LDL pre-treated with up to 2500 μM OCN^-^ did not significantly affect LAL activity in cell lysates (data not shown). In intact J774A.1 cells, LDL modified by HOSCN or HOCl for 30 min did not have a significant effect on LAL activity, whereas a significant decrease in LAL activity was observed on exposure of the cells to LDL pre-treated for 24 h with 250 μM HOSCN, 250 μM HOCl, and 2500 μM OCN^-^ compared to cells incubated with control LDL (Fig 8C).

## Discussion

Lysosomes contain a battery of enzymes that degrade internalised proteins and lipids, which enable metabolism of native LDL, and removal of potentially toxic particles such as modified LDL, from cells [6]. In this study, we show that the exposure of macrophages directly to HOCl and HOSCN, or to LDL modified by these oxidants, decreases the activity of the lysosomal, Cys-dependent, cathepsin enzymes B and L. These enzymes play a key role in the catabolism of proteins, and have been strongly implicated in the development of cardiovascular disease, with a deficiency in the expression and inhibition of cathepsins linked with foam cell formation [3, 36]. We also show that treatment of macrophages with modified LDL decreases the activity of the major lysosomal lipase, LAL, which is responsible for the hydrolysis of cholesteryl esters from LDL, and has also been implicated as contributing to the development of atherosclerosis [37].

It has been shown in this study that both cathepsins B and L are intracellular targets for HOSCN and HOCl, with the concentrations necessary to induce inactivation found to be similar for both oxidants, although these cells consume significantly less HOSCN compared to HOCl [34]. The concentration of HOCl and HOSCN required to cause cathepsin inactivation is within the patho-physiological range estimated to be formed *in vivo*, particularly under chronic inflammatory conditions, where local concentrations of up to 5 mM HOCl have been reported [38]. The amount of HOSCN produced *in vivo* is likely to be lower than HOCl, as the formation of this oxidant is limited to the concentration of SCN^-^, which is typically 50–120 μM in the plasma [39].

These data reflect the greater selectivity of HOSCN compared to HOCl for Cys residues [39], which are critical to the activity of these enzymes. This conclusion is supported by the observation that HOSCN-induced cathepsin inactivation could be reversed by DTT addition, which is consistent with the formation of Cys-derived oxidation products, such as sulfenic acids, as observed previously in macrophages exposed to this oxidant [40]. No evidence was obtained for oxidant-induced inactivation of cathepsin D, which has similar considerable sequence homology to cathepsins B and L, but has an Asp residue, rather than Cys in its active site, which is not reactive with HOSCN [39].

The Cys-dependent cathepsins B and L were also a target for LDL that had been modified by HOCl, HOSCN and the decomposition product OCN^-^. In the experiments with macrophage cell lysates, in general a more pronounced decrease in enzyme activity was seen when the LDL was pre-treated with HOCl or HOSCN for 30 min rather than 24 h. An exception to this was seen in lysates exposed to HOSCN-LDL, where similar loss in cathepsin B activity was observed at each incubation time. Overall, this difference is attributed to the formation of reactive species on the LDL, including *N*-chloramines in the case of HOCl [23], which are known to target intracellular thiol-containing enzymes [41, 42]. These data are consistent with a previous study showing that HOCl-modified LDL is capable of reducing isolated cathepsin B enzyme activity by a pathway involving *N*-chloramines formed from Lys residues present in the apoB100 protein [20]. Exposure of LDL to OCN^-^, which results in carbamylation of multiple residues, and the formation of homocitrulline (HCit) from Lys, also decreased cathepsin enzyme activity in the cell lysates. In this case, the mechanism is not certain, but may involve reversible carbamylation of Cys residues on LDL [43], and subsequent trans-carbamylation of the cathepsin B and L Cys residues by either these species directly, or via release of OCN^-^ from the LDL under the acidic conditions present in the lysosomal compartment.

A decrease in lysosomal cathepsin activity was also observed on exposure of intact macrophages to each type of modified LDL. The most pronounced decrease in enzymatic activity is seen with LDL pre-treated with HOCl or HOSCN for 24 h rather than 30 min, which results in the formation of more extensively modified LDL particles [23]. In addition, a greater extent of cathepsin B and L inactivation was seen with HOSCN-modified LDL, rather than HOCl-modified LDL. This was unexpected, as HOCl induces more widespread and extensive modification of the apoB100 protein compared to HOSCN, with this resulting in greater cellular uptake of the modified LDL as assessed by the accumulation of cholesterol and cholesteryl esters in both murine and human macrophages [23]. However, whilst HOCl targets almost exclusively the apoB100 protein [44], a greater extent of cholesterol and cholesteryl ester oxidation is seen on exposure of LDL to HOSCN or a MPO/H_2_O_2_/SCN^-^ system, which results in the formation of various products including conjugated dienes, lipid hydroperoxides, 9-HODE and F_2_-isoprostanes [23, 45]. The formation of these materials, including reactive aldehydes and hydroperoxides, may be responsible for the observed enzyme inhibition, with both these reactive species having been shown previously to inactivate Cys-dependent cathepsins [8, 32, 46]. A similar, though not as extensive, pattern of inactivation has been seen with LDL exposed to Cu^2+^ ions, which results in a modified particle capable of inactivating lysosomal enzymes, including cathepsin B [8, 46].

No changes in the expression of cathepsins B and L or lysosomal number were observed on exposure of macrophages to modified LDL, which indicates that the loss of cathepsin activity is not due to a decrease in protein expression or a reduction in lysosomal number. It has been proposed in previous studies with Cu^2+^-modified LDL that inactivation of cathepsin B occurs via the formation of covalent complexes mediated by reactive aldehydes, such as 4-hydroxynoneal (HNE), that form on decomposition of lipid hydroperoxides, which are liberated from the oxLDL under the acidic conditions prevalent in the lysosomal compartment [46]. It is not known whether exposure of LDL to HOSCN results in HNE formation, though evidence has been obtained for extensive lipid hydroperoxide formation, suggesting that this pathway could be involved in the cathepsin B (and L) inactivation. This pathway may also be applicable to HOCl-modified LDL, as inactivation of cathepsins B and L was only observed on treatment of the LDL with high (> 250 μM) oxidant concentrations, where lipid hydroperoxide formation, albeit at low concentration, has been shown to occur [23].

Evidence has also been obtained for decreased LAL activity in macrophages exposed to LDL modified by HOCl, HOSCN and OCN^-^. As with cathepsins B and L, the changes in LAL activity in intact cells were only observed on prolonged (24 h) incubation following exposure of the LDL to each oxidant. In this case, there was no significant difference between the extent of enzyme inactivation between treatment time of the LDL with oxidant or with each type of modified LDL. This may reflect the differences in the active site of LAL, which contains a critical Ser residue [47], which is more resistant to oxidation and / or modification compared cathepsins B and L, whose activities are dependent on active site Cys residues. Although it has been shown previously that lipid hydroperoxides have an inhibitory effect on cholesteryl esterases [48], this may not be the predominant mechanism involved in LAL inhibition in macrophages exposed to HOCl, HOSCN or OCN^-^ modified LDL, as a similar extent of LAL inactivation was observed with each type of LDL, and lipid hydroperoxide formation is only prevalent on HOSCN-modified LDL [23].

In the intact cell experiments, the loss in LAL activity may be related to a decrease in lysosomal acidity, resulting from the accumulation of free cholesterol and cholesteryl esters in the lysosomal membrane, as reported previously in THP1 macrophages exposed to Cu^2+^-modified LDL [49]. The increase in pH resulting in loss of LAL activity, which has an optimum pH of 4, has been attributed to the inhibition of the vacuolar H^+^-ATPase proton pumping activity in the lysosomal membrane in response to cholesterol loading within the macrophages [49]. Similar lysosomal pH effects have been reported in mouse peritoneal macrophages exposed to oxLDL and in macrophages isolated from the lesions of atherosclerosis-prone apoE^-/-^ mice [5]. The decrease in LAL activity may also be related to a decreased integrity of the lysosomal membrane, and a resulting decrease in the proton gradient [37]. Although there was no significant change in the protein levels of lysosomal cathepsins or LAMP-1 on exposure of the macrophages to each type of modified LDL, these experiments were performed with whole cell lysates, rather than lysosomal and cytosolic fractions.

In addition to the oxidation of lysosomal enzymes as a pathway to defective LDL detoxification, there are genetic lysosomal storage diseases, including Wolman disease and Niemann-Pick Type C (NPC) cholesterol storage disorder that also cause alterations in cholesterol processing and disruptions to intracellular lipid transport [50, 51]. Wolman disease, which is characterised by LAL deficiency, is associated with accelerated lesion development in atherosclerosis [52]. Similarly, the disruption of intracellular lipid transport and accumulation of lysobisphosphatic acid (LBPA) to compensate for the excess cholesterol in the late endosomes and lysosomes, is strongly linked with obesity, which is a key risk factor for cardiovascular disease [51, 53].

## Conclusions

We have shown that exposure of macrophages to HOCl and HOSCN or LDL modified by these MPO-derived oxidants results in altered lysosomal enzyme function, which is likely to reduce both proteolytic capacity and decrease cholesteryl ester hydrolysis. In the case of the lysosomal cathepsins B and L, the loss in activity is attributed to the modification of the active site Cys residues. With LAL, the loss in activity may be related to either the accumulation of reactive products (aldehydes or hydroperoxides) derived from the modified LDL, or cholesterol accumulation within the macrophages resulting in altered lysosomal pH. Although there are some limitations with extrapolating data obtained from experiments performed with the J774.A1 macrophage-like cell line, taken together, these results provide a potential pathway to help rationalise the accumulation of protein and lipids seen within the arterial wall during lesion development in atherosclerosis.
